# Patient participation in clinical trials conducted by principal investigators who speak one or more language(s) beyond english: Exploring ethnicity as proxy for language

**DOI:** 10.1016/j.conctc.2024.101353

**Published:** 2024-08-17

**Authors:** Anne Rivelli, Osondi Ozoani-Lohrer, Cheryl Lefaiver, Maureen Shields, Andy Marek, Mercedes Robaina, Veronica Fitzpatrick

**Affiliations:** a Advocate Aurora Research Institute, Milwaukee, WI, USA; b Advocate Health, Milwaukee, WI, USA; c Center for Child and Family Research, Milwaukee, WI, USA

**Keywords:** Ethnicity, Disparity, Clinical trial participation, Cultural sensitivity, Diversity

## Abstract

**Background:**

To explore the association between ethnicity, as a proxy for language, and participation in clinical trials (CT) conducted by Principal Investigators (PI) who speak one or more language in addition to English.

**Methods:**

This retrospective, descriptive study utilized CT participant demographic data extracted from the largest Midwestern non-profit healthcare system between January 1, 2019 and 12/31/2021. The CT participant sample (N = 4308) was divided for comparison: CT Participants of Hispanic or Latino Origin (N = 254; 5.90 %) and CT Participants of Non-Hispanic or Latino Origin (N = 4054; 94.10 %). Logistic regressions were performed to generate the crude and adjusted odds of patients of Hispanic or Latino origin participating in CTs conducted by PIs who speak another language in addition to English.

**Results:**

Crude analysis revealed that patients of Hispanic or Latino ethnicity had 2.04 (1.58, 2.64) times greater odds of participating in CTs conducted by PIs who speak another language than English (<0.0001), which increased to 2.67 (1.97, 3.62) times greater odds after adjusting for sex, race, age and insurance (p < 0.0001).

**Conclusions:**

Overall findings indicate that patients of Hispanic or Latino ethnicity, who are more likely to speak Spanish, have greater odds of participating in CTs conducted by PIs who speak another language beyond English. This may imply that cultural sensitivity at the top of a CT study team, as likely to be demonstrated by PIs who speak another language beyond English, may be an important contributor to reducing ethnicity- and language-based barriers to diversity in CTs and a relationship worth exploring further.

## 1 Introduction

There is a need for adequate representation of minority populations in clinical trials (CTs) to ensure generalization of results, learn about potential differences among groups, and improve health outcomes for all individuals [1,2]. However, there are persistent structural impediments to enrolling representative samples of racially and ethnically minoritized individuals into CTs [[3], [4], [5]]. Poor recruitment of minoritized populations in research is complex but can be attributed to system factors such as insufficient recruitment strategies and planning [5,6], lack of cultural competency among researchers [5,7], and poor engagement and linkages with communities and individuals [8,9], as well as patient factors like historically poor experience of participating in research and communication issues [7]. Communication issues, specifically low literacy and language differences, have all been cited as common barriers to enrollment of minority populations into CTs [7,[10], [11], [12]].

Language barriers present a unique challenge to diverse clinical practices [7,13,14]. Studies suggest that the language spoken by patients and research professionals may be more important of a factor in enrollment than race [[15], [16], [17]], not only that a patient's primary language influenced enrollment success, but research professionals had more success enrolling subjects who shared their primary language (“language concordance”) [15] Further, a majority indicated that the language barrier and time spent arranging for interpreters had prevented them from offering a study to potential candidates [15,16]. While it might be intuitive that a CT investigator being the same race and/or ethnicity as a potential study subject (“racial concordance”) might appear to be motivating to minorities' participation studies show that race and ethnicity of potential candidates did not always indicate they were more successful in enrolling subjects of their own race for various reasons [15,[18], [19], [20], [21]]. While it is highly unlikely to have research professionals speak every patient language without interpreter services, multicultural research staff may improve representation in CT enrollment by potentially having more positive attitudes related to cultural differences among potential participants. Given that language barriers tend to be one of the less surmountable challenges to enrollment [15], this paper will explore the hypothesis that CT principal investigators (PIs) who speak one or more additional languages beyond English (proxy for multiculturalism), may have greater participation of eligible patients who speak a language other than English even though there may not be PI-patient language concordance [22].

There is abundant research documenting the under-representation of patients identifying as Hispanic or Latino ethnicity, specifically [[23], [24], [25]]. What seems less clear is potential differences in characteristics of patients of Hispanic or Latino origin participating in CTs as compared to patients not of Hispanic or Latino origin and, further, as compared to patients of Hispanic or Latino origin being treated by CT PIs overall, representing potential eligible (but non-participating) participants. We suspect language is a significant contributing factor to enrollment of patients of Hispanic or Latino origin, given the additional time and effort required for a language interpreter.

The objective of this paper to compare the proportion of participants of Hispanic or Latino origin in CTs conducted by PIs who speak two or more languages (proxy for multiculturalism), to proportions of participants of Hispanic or Latino origin in CTs conducted by PIs who speak English only. Capturing language preferences in a diverse healthcare system is not without challenges, primarily being that a substantial number of patients will have their documented preferred language as English, even if it is not, making it a barrier for researchers to quantify patient-provider language concordance. Given this constraint, ethnicity is used as a proxy for Spanish speaking, given that 75 % of people identified as Hispanic speak Spanish and this is increased to 93 % if foreign born [26]. In the Chicago metropolitan area, where these data derive from, nearly 2 million people speak Spanish and is greater than the national average for urban areas. Additionally, 25 % of the Chicagoland area speaks a language other than English but unfortunately in our clinical trials data management system ‘language’ is not accurate enough to use in this study [27].

## 2 Materials and methods

This retrospective, descriptive study focused on differences by ethnicity utilizes patient demographic data from two groups extracted from the Clinical Trials Management System (CTMS) and electronic medical record (EMR) within the largest Midwestern non-profit healthcare system. The dataset includes data from a sample of 4308 unique healthcare system patients who provided informed consent to participate in at least one active CT within the system between January 1, 2019 and December 31, 2021 (“CT Participants”). The original dataset included 4321 unique CT participants but was limited to the 4308 patients who had ethnicity and PI data in the timeframe, reflecting 0.30 % missing participant data which the research team deemed small enough to proceed with a valid analysis.

### 2.1 Data

Patient characteristics were collected from CTMS and EMR on the CT Participants and the CT Patients of Hispanic or Latino Origin, including: Ethnicity (categorical), Hispanic or Latino, Non-Hispanic or Latino; Sex (categorical), Male or Female; Race (categorical), White, Black, Asian, American Indian or Alaskan Native, Native Hawaiian and Other Pacific Islander; Insurance type (categorical), Private, Medicare, Medicaid, Self-pay, Worker's Comp; and date of birth to calculate age (continuous) as of January 1, 2019 for both groups and categorize into groups. Patient characteristics were captured from CTMS at clinical trial enrollment in the timeframe (January 1, 2019–December 31, 2021). Gender and languages spoken were collected on all PIs of CTs active from January 1, 2019 and December 31, 2021 from the publicly-available healthcare website.

### 2.2 Statistical methods

Data management and analysis of the sample were conducted with SAS statistical software (Version 9.4; SAS Institute, Cary, NC). All analyses of the sample data were performed by research personnel employed by the health system. Proportions with binomial exact 95 % confidence intervals were calculated across all demographic variable levels across both participant groups and the prospective participants of Hispanic or Latino origin. Pearson chi-square tests were performed to evaluate differences by ethnicity, overall and stratified by patient characteristics, in participation in studies conducted by PIs who spoke at least one other language than English. Fisher's Exact Test p-values were interpreted for variables with at least one cell with a value less than 5 (as indicated by ^ below). Crude logistic regressions were conducted to determine associations between patient characteristic levels and the outcome of participation in CTs conducted by PIs who speak at least one other language beyond English. Finally, a logistic regression was conducted to determine the adjusted association between ethnicity and participation in CTs conducted by PIs who speak at least one other language beyond English, adjusting for all patient characteristics, specifically sex, race, age and insurance.

## 3 Results

CT Participants (N = 4308) were divided into two groups: CT Participants of Hispanic Origin (N = 254; 5.90 %) and CT Participants of Non-Hispanic Origin (N = 4054; 94.10 %). Characteristics overall and by ethnicity, specifically CT Participants of Hispanic or Latino Origin and Non-Hispanic or Latino Origin, are described in detail in Table 1. Generally, fewer females of Hispanic or Latino origin participated relative to females of Non-Hispanic or Latino origin while, conversely, more males of Hispanic or Latino origin participated relative to males of Non-Hispanic or Latino origin. Generally, more patients of Hispanic or Latino origin who were White, American Indian/Alaskan Native and Native Hawaiian/Other Pacific Islander participated relative to patients of Non-Hispanic or Latino origin in these racial groups. Fewer patients of Hispanic or Latino origin who were Black and Asian participated relative to patients of Non-Hispanic or Latino origin in these racial groups. Generally, more younger patients who were of Hispanic or Latino origin participated relative to those who are of Non-Hispanic or Latino origin. Further, while proportions of patients of Hispanic or Latino origin participating in CTs increased with age, the gap in participation relative to patients of Non-Hispanic or Latino origin shifts, with more younger patients of Hispanic or Latino origin and more older patients of Non-Hispanic or Latino origin participating. Generally, more patients who were of Hispanic or Latino origin with Medicaid insurance participated in CTs relative to those who are of Non-Hispanic or Latino origin, while less patients of Hispanic or Latino origin with Medicare insurance participated relative to those of Non-Hispanic or Latino origin. Patients with Private, Self-Pay and Worker's Compensation insurances participated fairly.

**Table 1:** Demographic Distributions of CT participant sample, overall and by ethnicity, representing a timeframe of January 1, 2019 to December 31, 2021.

Patient Characteristics	*All CT Participants* N = 4308	*CT Participants of Hispanic or Latino Origin* N = 254 (5.90 %)	*CT Participants of Non-Hispanic or Latino Origin* N = 4054 (94.10 %)
*Sex*			
Female	2459 (57.08 %)	135 (53.15 %)	2324 (57.33 %)
Male	1849 (42.92 %)	119 (46.85 %)	1730 (42.67 %)
*Race*	*N = 4271*	*N = 231*	*N = 4040*
AI/AN	22 (0.52 %)	5 (2.16 %)	17 (0.42 %)
Asian	85 (1.99 %)	1 (0.43 %)	84 (2.08 %)
Black	515 (12.06 %)	7 (3.03 %)	508 (12.57 %)
NH/PI	11 (0.26 %)	7 (3.03 %)	4 (0.10 %)
White	3638 (85.18 %)	211 (91.34 %)	3427 (84.83 %)
*Age Category*			
18.00 < 29.00	295 (6.85 %)	44 (17.32 %)	251 (6.19 %)
29.01 < 39.00	387 (8.98 %)	29 (11.42 %)	358 (8.83 %)
39.01 < 49.00	381 (8.84 %)	39 (15.35 %)	342 (8.44 %)
49.01 < 59.00	714 (16.57 %)	44 (17.32 %)	670 (16.53 %)
59.01 < 69.00	1161 (26.95 %)	45 (17.72 %)	1116 (27.53 %)
69.01 < 79.00	982 (22.79 %)	36 (14.17 %)	946 (23.33 %)
≥79.01	388 (9.01 %)	17 (6.69 %)	371 (9.15 %)
*Insurance*			
Medicaid	361 (8.38 %)	50 (19.69 %)	311 (7.75 %)
Medicare	2234 (51.86 %)	96 (37.80 %)	2138 (53.26 %)
Private	1485 (34.47 %)	91 (35.83 %)	1394 (34.73 %)
Self-Pay	177 (4.11 %)	15 (5.91 %)	162 (4.04 %)
Worker's Comp	11 (0.26 %)	2 (0.79 %)	9 (0.22 %)

**Fig. 1:**
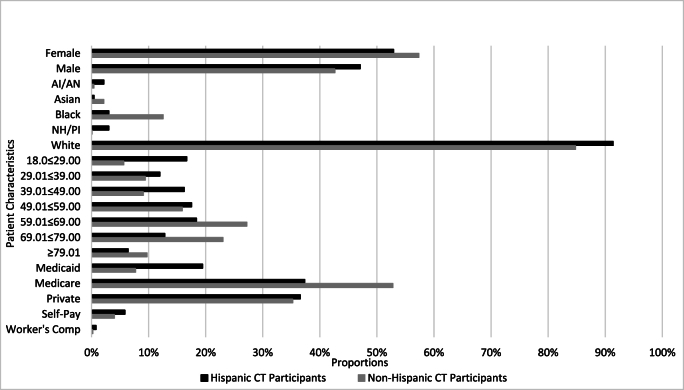
Patient Characteristics of Hispanic or Latino Participants vs. Non-Hispanic or Latino Participants.

Equally regardless of ethnicity. See Fig. 1 for a visual display of these characteristics.

In the study timeframe, there were 130 total healthcare system providers serving as PIs of active CTs, 86 (78.90 %) of which were male, 23 (21.10 %) female and 21 (16.15 %) missing gender. Among all PIs, 88 (67.69 %) spoke only English and 42 spoke at least one additional language, specifically24 (18.46 %) spoke two languages (one in addition to English), 17 (13.08 %) spoke three languages (two in addition to English), and 1 (0.77 %) spoke four languages (three in addition to English). Overall, the 42 PIs spoke 23 different languages beyond English See Table 2 for available provider details and Table 3 for a breakdown of all languages spoken by the 130 PIs included in this study.

**Table 2:** Demographics of clinical trial principal investigators serving CT participants and CT patients of hispanic or latino origin from January 1, 2019 to December 31, 2021.

Provider Characteristics	*Healthcare System Providers/CT PIs* N = 130
*Sex*	
Female	23 (21.10 %)
Male	86 (78.90 %)
Languages	
1 (English Only)	88 (67.69 %)
2	24 (18.46 %)
3	17 (13.08 %)
4	1 (0.77 %)

**Table 3:** Languages spoken by PIs.

English	130 (100.0 %)
Hindi	12 (9.2 %)
Spanish	9 (6.9 %)
Urdu	9 (6.9 %)
Arabic	4 (3.1 %)
French	3 (2.3 %)
Gujarati	3 (2.3 %)
German	2 (1.5 %)
Punjabi	2 (1.5 %)
Russian	2 (1.5 %)
Tamil	2 (1.5 %)
Amharic	1 (0.8 %)
Greek	1 (0.8 %)
Hebrew	1 (0.8 %)
Igbo	1 (0.8 %)
Korean	1 (0.8 %)
Mandarin	1 (0.8 %)
Persian	1 (0.8 %)
Portuguese	1 (0.8 %)
Romanian	1 (0.8 %)
Sinhala	1 (0.8 %)
Swedish	1 (0.8 %)
Telugu	1 (0.8 %)
Turkish	1 (0.8 %)

Pearson chi-square tests were conducted to explore associations between ethnicity and odds of participating in CTs conducted by PIs who speak another language than English, overall and stratified by each available patient characteristic. Relative to those who were of Non-Hispanic or Latino ethnicity, patients of Hispanic or Latino ethnicity overall and by most characteristics (female and male sex, Black and White race, all age categories, and all insurance types) were associated with greater odds of being participants in CTs conducted by PIs who spoke at least one other language than English by statistically and/or clinically significant odds ranging between 1.20 (0.59, 2.42) and 5.24 (2.62, 10.47).

**Fig. 2:**
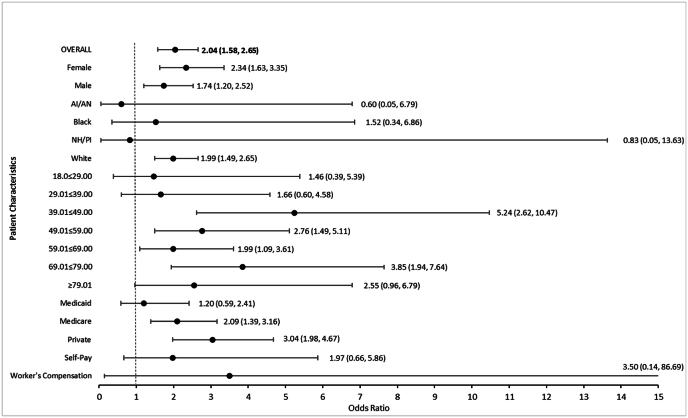
Overall and stratified findings Reveal patients of hispanic or latino ethnicity have greater odds of participating in clinical trials conducted by principal investigators who speak another language than English.

Relative to those who were not of Hispanic or Latino ethnicity, patients of Hispanic or Latino ethnicity who were American Indian or Alaskan Native (AI/AN) race or Native Hawaiian or Other Pacific Islander (NH/PI) were associated with lesser odds of being participants in CTs conducted by PIs who spoke another language than English, with clinically significant odds of 0.60 (0.05, 6.79; p = 1.0000^) and 0.83 (0.05, 13.63; p = 1.0000^), respectively. Odds could not be generated for patients of Asian race due to a cell count of zero. See Fig. 2 for forest plots visualizing all relative odds.

Next, logistic regression analyses were then performed to look at the crude and adjusted associations between ethnicity and participation in a CT run by a PI who spoke more than just English. Crude analysis revealed that patients of Hispanic or Latino ethnicity had 2.04 (1.58, 2.64) times greater odds of participating in CTs conducted by PIs who speak another language than English (<0.0001). After adjusting for sex, race, age and insurance, adjusted analysis revealed that patients of Hispanic or Latino ethnicity had 2.67 (1.97, 3.62) greater odds of participating in CTs conducted by PIs who speak another language than English (p < 0.0001). This increase from 2.04 in the crude model to 2.67 greater odds in the adjusted model indicates that controlling for other patient characteristics strengthens the true association between ethnicity and participation in CTs conducted by PIs who speak more than English. See Table 4 for a complete report of crude and adjusted parameter estimates and p-values.

**Table 4:** Crude and adjusted parameter estimates of patient characteristics associated with participation in clinical trials conducted by principal investigators who speak a language beyond English.

	Crude Models			Final Adjusted Model		
Model Parameters	β (SE)	Odds Ratio (OR)	P-value	β (SE)	Odds Ratio (OR)	P-value
*Intercept*	–	–	–	−0.36 (0.24)	–	0.1432
**Patient Characteristics**						
*Ethnicity*						
Non-Hispanic or Latino	−0.68 (0.07)	1.00	<0.0001	REF		
Hispanic or Latino	0.32 (0.07)	2.04 (1.58, 2.64)	<0.0001	**0.49 (0.08)**	**2.67 (1.97, 3.62)**	**<0.0001**
*Sex*						
Male	−0.93 (0.03)	1.00	<0.0001	REF		
Female	−0.34 (0.03)	0.52 (0.45, 0.59)	<0.0001	−0.29 (0.04)	0.56 (0.48, 0.64)	<0.0001
*Race*						
White	−0.36 (0.17)	1.00	0.0381	REF		
American Indian or Alaskan Native	−0.62 (0.41)	1.06 (0.41, 2.71)	0.1280	−0.73 (0.42)	0.96 (0.36, 2.53)	0.0868
Asian	0.29 (0.24)	2.62 (1.69, 4.05)	0.2383	0.61 (0.26)	3.65 (2.80, 5.84)	0.0181
Black	−0.33 (0.19)	1.42 (1.16, 1.73)	0.0821	−0.01 (0.20)	1.95 (1.57, 2.43)	0.9557
Other Pacific Islander	1.34 (0.55)	7.50 (1.99, 28.34)	0.0153	0.81 (0.59)	4.43 (1.07, 18.39)	0.1725
*Age*						
18.00–29.00	−1.30 (0.05)	1.00	<0.0001	REF		
29.01–39.00	−0.72 (0.14)	2.42 (1.32, 4.43)	<0.0001	−0.74 0.15)	2.81 (1.50, 5.27)	<0.0001
39.01–49.00	0.32 (0.11)	6.87 (3.90, 12.10)	0.0038	0.20 (0.12)	7.16 (3.94, 13.03)	0.1015
49.01–59.00	0.54 (0.09)	8.57 (4.98, 14.76)	<0.0001	0.53 (0.09)	9.93 (5.60, 17.63)	<0.0001
59.01–69.00	0.69 (0.07)	9.94 (5.83, 16.94)	<0.0001	0.73 (0.08)	12.22 (6.85, 21.81)	<0.0001
69.01–79.00	0.47 (0.08)	7.94 (4.64, 13.59)	<0.0001	0.61 (0.10)	10.81 (5.94, 19.67)	<0.0001
79.01+	0.31 (0.11)	6.76 (3.84, 11.91)	0.0057	0.43 (0.13)	9.05 (4.83, 16.96)	0.0007
*Insurance*						
Private	−1.04 (0.14)	1.00	<0.0001	REF		
Medicaid	−0.27 (0.17)	0.77 (0.58, 1.03)	0.1237	−0.00 (0.19)	0.87 (0.64, 1.19)	0.9993
Medicare	0.21 (0.15)	1.28 (1.10, 1.48)	0.1566	−0.05 (0.17)	0.83 (0.67, 1.02)	0.7536
Self-Pay	0.02 (0.19)	1.06 (0.74, 1.51)	0.9195	0.01 (0.21)	0.88 (0.60, 1.29)	0.9556
Worker's Compensation	0.06 (0.54)	1.10 (0.29, 4.17)	0.9177	−0.10 (0.60)	0.79 (0.18, 3.42)	0.8723

## 4 Discussion

This study was performed using two EMR data sources within a large Midwestern U.S. health system, one including all patients who participated in an active CT between January 1, 2019 and December 31, 2021 and another including only patients of Hispanic or Latino ethnicity treated by the PIs of all active CTs between January 1, 2019 and December 31, 2021. In this paper, ethnicity was a key variable of interest to represent diversity on a broader scale, but also to serve as a proxy for primary language. Primary language or perceived primary language may be an important factor associated with poor participation in CTs overall but, given true primary language may be inaccurately documented in medical records [28,29], using ethnicity as a proxy is a first, albeit imperfect, step.

Further, the outcome of participation in CTs conducted by PIs who speak one or more languages beyond English in this study is intended as a proxy for multiculturalism to include cultural sensitivity and respect for others’ culture. This research team pursued a hypothesis that PIs and/or research teams led by PIs who were more diverse and more aware of and sensitive to different cultures, as defined by speaking one or more languages beyond English, is associated with recruitment of and/or participation among patients who were Hispanic or Latino.

One objective of this paper is to explore associations between ethnicity and an outcome of participation in CTs conducted by PIs who speak language(s) beyond English, overall and stratified by patient characteristics, for a close look at potential differences by ethnicity within each patient characteristic. Patients of Hispanic or Latino ethnicity had two times greater odds of participating in CTs conducted by PIs who speak another language than English, which introduces a novel area for future research. This study also found more Hispanic or Latino patient participation, clinically if not just statistically, in CTs conducted by PIs who spoke more than just English across most characteristics – female, male, black, white, all age categories, and all insurance categories. It is important to point out that only 6.9 % of PIs spoke Spanish, so there is likely very little language concordance among Hispanic or Latino participants with their PI. These initial findings show the association between ethnicity and participation in CTs conducted by more culturally sensitive PIs was worth exploring.

Another objective of this paper is to determine the association between ethnicity and participation in CTs conducted by PIs who speak language(s) beyond English through a logistic model adjusting for all patient characteristics. After adjusting for all patient characteristics, patients of Hispanic or Latino origin had closer to 3 times greater odds of participating in CTs conducted by PIs who spoke another language than English. After controlling for other demographics that may contribute to greater participation in CTs by PIs who speak more than just English, patients identifying as Hispanic or Latino show much greater odds of being participants.

It should be noted that this study, with the currently available and approved data, does not and cannot assess all invitations or eligible to participate in CTs. To this end, it is possible that this relationship could be explained by patients of Hispanic or Latino ethnicity more often seeing providers who speak more than just English for medical care and, thus, were more susceptible to invitation and participation to participate in CTs conducted by such PIs; however, only 9 (6.92 %) of all 130 PIs represented in this study spoke Spanish as an additional language and any other reasoning for this potential bias is unknown and unsuspected.

Overall findings indicate that patients of Hispanic or Latino ethnicity, who are more likely to speak Spanish, have greater odds of participating in CTs conducted by PIs who speak another language beyond English. This may imply that cultural sensitivity at the top of a CT study team, as likely to be demonstrated by PIs who speak another language beyond English, may be an important contributor to more diverse participation in CTs. While the findings of this study cannot conclude this with certainty, our results suggest this is a possibility worth exploring further. These findings do suggest that concordance related to some element of diversity or trained cultural sensitivity may be a valuable factor in reducing language-based barriers to diversity in CT participation. Even so, further evidence of this relationship will not solve the logistical barriers, like need for interpreters, that may contribute to lack of diverse participation in CTs, like the use of interpreters.

### 4.1 Future research

While this study chose to focus on patients of Hispanic or Latino ethnicity who are most likely to speak the Spanish language, future research with appropriate patient samples should broaden this to other patient languages such as any of the Asian origin languages. Further, it should be noted that numbers of patients across Non-White racial categories are curiously low. It is generally accepted that documentation in healthcare of sensitive data like race is poor and often based on external appearance assumptions, but it would be of a great value to explore the race-stratified association between ethnicity and participation in CTs conducted by PIs who speak language(s) beyond English with larger numbers of patients in different racial groups.

## Funding

This work was supported by the Advocate Charitable Foundation.

## Ethics approval

This study was approved by the healthcare system's Institutional Review Board (#22.017E).

## Consent to participate

Written consent from participants was not obtained. For practicality reasons, the Institution Review Board approved a waiver of consent/authorization for this study.

## CRediT authorship contribution statement

**Anne Rivelli:** Writing – review & editing, Writing – original draft, Validation, Methodology, Investigation, Formal analysis, Data curation, Conceptualization. **Osondi Ozoani-Lohrer:** Writing – original draft, Project administration. **Cheryl Lefaiver:** Writing – review & editing, Project administration, Investigation, Data curation, Conceptualization. **Maureen Shields:** Writing – review & editing, Project administration, Data curation, Conceptualization. **Andy Marek:** Data curation. **Mercedes Robaina:** Writing – review & editing, Project administration, Funding acquisition, Conceptualization. **Veronica Fitzpatrick:** Writing – review & editing, Writing – original draft, Project administration, Methodology, Investigation, Data curation, Conceptualization.

## Declaration of competing interest

The authors declare that they have no known competing financial interests or personal relationships that could have appeared to influence the work reported in this paper.
